# Congenital syphilis in East Baton Rouge parish, Louisiana: providers’ and women’s perspectives

**DOI:** 10.1186/s12879-020-05753-6

**Published:** 2021-01-13

**Authors:** Emily W. Harville, Gloria P. Giarratano, Pierre Buekens, Eurydice Lang, Jennifer Wagman

**Affiliations:** 1 Department of Epidemiology, Tulane School of Public Health and Tropical Medicine, 1440 Canal St. #8318, New Orleans, LA 70112 USA; 2 School of Nursing, Louisiana State University Health Sciences Center, New Orleans, LA 70112 USA; 3 Department of Community Health Sciences, UCLA Fielding School of Public Health, Los Angeles, CA 90095-1772 USA; 4 Division of Infectious Diseases and Global Public Health, Department of Medicine, School of Medicine UC San Diego, San Diego, CA 92093 USA

**Keywords:** Syphilis, congenital, Prenatal care, Social determinants of health, Qualitative methods

## Abstract

**Background:**

Congenital syphilis is completely preventable through screening and treatment, but rates have been rising in the United States. Certain areas are at particularly high risk. We aimed to assess attitudes, knowledge, and barriers around effective prevention of congenital syphilis among health care providers and community women potentially at risk.

**Methods:**

Two parallel studies were conducted: in-depth interviews with health care providers and focus groups with community women in the area of Baton Rouge, Louisiana. Each group was questioned about their experience in providing or seeking prenatal care, knowledge and attitudes about congenital syphilis, sources of information on testing and treatment, perceptions of risk, standards of and barriers to treatment. Results were transcribed into QSR NVivo V10, codes developed, and common themes identified and organized.

**Results:**

Providers identified delays in testing and care, lack of follow-through with partner testing, and need for community connection for prevention, as major contributors to higher rates of congenital syphilis. Women identified difficulties in accessing Medicaid contributing to delayed start of prenatal care, lack of transportation for prenatal care, and lack of knowledge about testing and prevention for congenital syphilis.

**Conclusions:**

Providers and community members were in broad agreement about factors contributing to higher rates of congenital syphilis, although some aspects were emphasized more by one group or another. Evidence-based interventions, likely at multiple levels, need to be tested and implemented to eliminate congenital syphilis.

**Supplementary Information:**

The online version contains supplementary material available at 10.1186/s12879-020-05753-6.

## Background

**Fig. 1:**
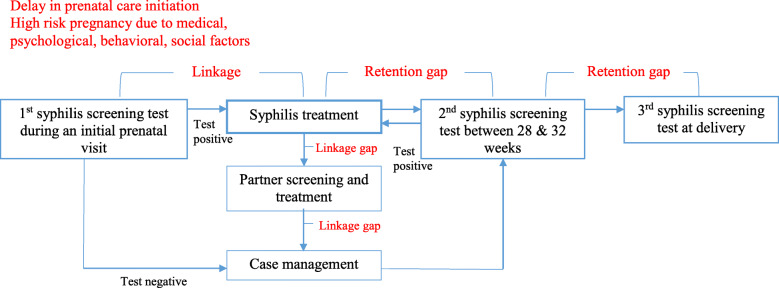
Congenital syphilis prevention cascade to identify linkage and retention gaps in high-risk pregnancy. Adapted from Park et al., under review

A dramatic increase in congenital syphilis was documented between 2012 and 2014 in all regions of the U.S. [1]. Theoretically, prevention of congenital syphilis is relatively simple and inexpensive. Screening all pregnant women for syphilis during antenatal care is feasible and the condition is treatable with antibiotics. In reality, however, barriers arise at the community, provider, program, and policy levels (Fig. 1). Women with limited health insurance or with substance use issues are at increased risk for inadequate prenatal care. Many cases of congenital syphilis result from lack of screening or screening and treatment too late for prevention of maternal-to-child transmission. For instance, 22% of the congenital syphilis cases documented in 2014 occurred in mothers who received no prenatal care, while 15% had accessed care but were not tested by their health provider [1].

Louisiana has one of the highest rates of congenital syphilis in the country- about four times the national average [2], and East Baton Rouge Parish was identified as one of the counties with the highest rates. In 2017, the case rate in Louisiana was 96 cases/100,000 livebirths, [3] while the national rate was 23.3 cases/100,000 livebirths [4]. The Louisiana STD/HIV/Hepatitis program estimated that while, overall, 94% of women were screened for syphilis during pregnancy, in some practices, this was as low as 66% [5]. A study of pregnant women who tested positive for syphilis in Louisiana and Florida in 2013–2014 found that 8% received insufficient treatment and 3% refused treatment [6]. These compare unfavorably with the World Health Organization’s targets of ≤50 cases/100,000 livebirths, ≥95% screening, and ≥ 95% treatment [7]. A 2014 investigation of Louisiana congenital syphilis cases and processes found that the majority of cases were identified via positive tests on the infant rather than the mother, suggesting that prenatal screening was ineffective or missing [8]. For this reason, the Centers for Disease Control and Prevention (CDC) and March of Dimes [9] partnered to examine attitudes, knowledge, and barriers to prevention and treatment in this area (as well as in Kern County, California). The study drew on knowledge-attitude-behavior theories for health education, [10] seeking to understand the baseline level of knowledge, sources of information, and decision-making, including constraints on those decisions. The qualitative study aimed to address these issues among both prenatal health care providers and pregnant women potentially at risk.

## Methodology

This qualitative inquiry is guided by the Social Ecological Model (SEM) adapted by Diclemente et al. [11, 12] to consider how multiple influencing factors contribute to a rise in congenital syphilis at individual, macroenvironment, and microenvironment levels. Two parallel methods of data collection were used: in-depth interviews with health care providers, and focus groups with community women. Consistent with SEM, the open-ended questions that guided interviews and focus group discussions were developed to guide study participants to reflect upon individual factors, personal/family/or professional relationships, and community and societal contexts that might have contributed to high congenital syphilis rates and what actions/changes within those systems can address this problem. All data was electronically recorded during the interview or group session and later transcribed verbatim to text for data analysis.

### In-depth interviews with health care providers

Eligibility criteria for participation in an in-depth interview were: (1) Prenatal care provider (including obstetricians and gynecologists, family physicians, maternal-fetal specialists, nurse-midwives, or nurse practitioners) who worked in East Baton Rouge Parish, Louisiana for at least 6 months and (2) Currently working directly with high-risk pregnant women. Participants were recruited from Woman’s Hospital, a large delivery hospital, and community clinics. The study was introduced at a staff meeting at Woman’s, while community clinics were contacted directly. Research nurses (GG and EL) conducted the in-depth qualitative interviews, using a semi-structured interview guide with a series of open-ended questions about the provider’s experience in providing prenatal care in the community; knowledge about and sources of information about congenital syphilis; perceptions of risk, standards of and barriers to treatment; and methods of communicating with patients (Script in supplementary materials). Interviews generally lasted one hour, and providers received a $50 gift card for participating.

### Focus groups with community women

Women were recruited from organizations in the Baton Rouge area that serve women who might be at high risk for congenital syphilis, such as women in poverty, or with a history of contact with incarceration, substance use, or homelessness. Family Road of Greater Baton Rouge is an agency that provides services to families in the Baton Rouge area, including coordinating enrollment in Medicaid, Women’s, Infants, and Children nutritional program (WIC), Supplemental Nutrition Assistance Program (SNAP), and Healthy Start. Family Road caseworkers informed clients of the focus groups, and recruiting flyers were posted. At a Residents’ Clinic at Woman’s, staff and providers at the clinic promoted recruitment into the study to their patients. Minimum eligibility criteria for focus group participants included: (1) Adult women (18 years and older) living in or receiving prenatal care in Baton Rouge Parish (County), Louisiana for at least 6 months; (2) Currently pregnant or had a baby less than 3 months old; (3) Having a phone or some other way of being contacted; and (4) English-speaking. There was no requirement of history of congenital syphilis or any other medical criteria to indicate high risk (such as other sexually transmitted diseases [STDs]).

Research nurses (EL and GG) conducted three focus groups of 8–15 pregnant and recently-pregnant women. Women were asked to consider pregnant women like themselves in the community but were not asked about their own experiences specifically. Open-ended questions covered health information seeking, use of health services, and knowledge and attitudes about congenital syphilis. The focus groups generally lasted 1 to 1 ½ hours, and women received a $25 gift card and transportation arrangements for participating.

### Analysis

Recorded data from the interviews and focus groups was transcribed into text. Researchers compared the transcribed text to the recordings and made corrections as needed. Data was imported into QSR NVivo V10 [13] and coded to identify health care provider and women’s perceived barriers to early and timely prenatal care, syphilis testing, and patient/community education that contributes to the incidence of syphilis. Coded text was extracted and organized [13] to identify emergent themes.

Both studies were approved by the Tulane and LSU Institutional Review Boards, and participants provided written informed consent.

## Results

Of the ten prenatal care providers interviewed (Table 1), eight were obstetricians and gynecologists, one a maternal-fetal-medicine specialist, and one a family nurse practitioner. Length of time in clinical practice ranged from one year to 37. Five (50%) of the prenatal providers worked in a state-supported clinic that trains obstetrical residents, and serves as a safety-net clinic in the community, accepting women at all stages of pregnancy, including those having difficulty finding a provider or who might be “dismissed” by a private provider for non-compliance issues. Providers described this clinic as mostly “all low-income women,” “90% African American”, with many high-risk medical and social conditions. Three providers worked in clinics with mixed low-income, Medicaid-insured and privately-insured women, including the maternal-fetal medicine specialist who accepted referrals from obstetricians regionally. Two provided prenatal care through a federally insured clinic in Baton Rouge that also served low income, underserved populations.

**Table 1:** Description of in-depth interview participants (health care providers)

Type of practice	
Single Specialty	80% (8)
Community/public health	20% (2)
Years in practice	
More than 5 years	50% (5)
1–5 years	50% (5)
Patients’ Primary Racial Background	
White or Caucasian	20% (2)
Black or African American	70% (7)
Hispanic or Latino	10% (1)
Patients’ Primary SES	
Lower Socio-economic	80% (8)
Middle Socio-economic	10% (1)
Higher Socio-economic	10% (1)

Each provider reported being well-aware of the rising numbers of STDs and congenital syphilis in Louisiana. All except one reported caring for a woman positive for syphilis in the past year. All the providers were knowledgeable of the recommended screening, testing, and treatment protocols to prevent congenital syphilis. Each referred to using both CDC and American College of Obstetricians and Gynecologists (ACOG) guidelines. Three mentioned having the “CDC app” [14] on their phone for easy access to STD care information in the clinic or hospital setting. Most obstetricians added that as an additional safety precaution, a final screen of all pregnant women for syphilis is done on admission for labor, regardless of previous screening.

### Prenatal care providers: theme: barriers to optimal care

Analysis of interview data revealed three sub-themes related to the overarching theme of “Barriers to optimal care”: (1) Delays in testing and care, (2) Lack of follow-through with partner testing, and (3) Need for community connection for prevention.

### Delays in testing and care

The providers often dealt with delays originating from patient or health care system barriers. For instance, although screening for syphilis is typically ordered at the first prenatal-patient encounter, some women enter care late.

*We have a lot of women that receive late care in our clinic, I’d say about 30% of them initiate their care* ***after*** *13-14 weeks into their pregnancy, well into their second trimester. And I’d say another 30% in their third trimester or no care at all. – OB/GYN, Residents’ Clinic*

Even the providers seeing women in private practices reported delayed prenatal care, particularly with Medicaid recipients.

*Probably about 30 to 40% enter prenatal care in the second trimester. USUALLY, that’s about the percentage of Medicaid patients I see. Usually by the time they get their Medicaid and their insurance straight, they’re in their second trimester... I would say it’s probably a smaller percentage that are past 20 weeks. -OB/GYN, Private Clinic*

Another provider also reported late entry times for many Hispanic women:

*It’s a higher number than you would think, particularly in the Medicaid clinic, and because this large Hispanic population that we take care of... We get them eligible for Medicaid—whatever sort of services they can get—and get them into our clinic. We see quite a few women who are WELL into their second or start of their third trimester. –OB/GYN, Private Clinic*

Another, less common, problem associated with delay occurred when women eluded the prenatal testing ordered. None of the prenatal provider clinics housed labs or provided phlebotomists; women were referred to a lab on a different floor or in an adjacent building. Occasionally, a woman chose not to go to the lab or get blood drawn and this would not be “caught” until the subsequent prenatal visit.

*… if they don't get their blood work done, they're gonna have a follow-up appointment. That is either addressed at the follow-up appointment, or in a perfect world we’d call and say “hey why don't you have your lab work done” but a lot of the times we don't know that they didn't have their lab work done until they come back for a follow-up.- OB/GYN, Residents’ Clinic*

Once testing is done, other potential delay points occurred in relaying a positive screen to the health provider, getting the message to the woman, and the woman returning for treatment and follow-up, although most care providers believed it to be rare not to eventually track down and have a pregnant woman to return for treatment.

*We usually get it [results] back either the same day or the next day. And we usually have to call them to come back in to get treated. And so some of them actually don’t return. -OB/GYN, Residents’ Clinic*

*Unfortunately, a challenge is getting ahold of our patients when they leave the office. We always try to contact them three times and then we send them a certified letter. Then we try and call their emergency contact. Any number I can get my hands on them.* -*OB/GYN, Residents’ Clinic*

*And so you’ll call them one day and it’s not working and call them the next day and it’s working, call them the next day and it’s not working again. And you call their emergency contact number and they’ll say “oh they’re staying right here and she’s out of minutes, her phone is off or she got a new phone … ” or they just get burner phones.* -*OB/GYN, Residents’ Clinic*

While all providers reflected their frustration in trying to both notify pregnant women with positive screens and get them to return for treatment, some were empathetic to the reasons for women’s difficulty in staying connected to health care.

*Part of it is trying to track down people, they have different residences. They may give you information, especially if there are drug related issues, that is false. They worry about testing positive for drugs, and then child protection taking their children so. There are a lot of factors that go on that make it difficult to track down people sometimes.- OB/GYN, Residents’ Clinic*

*I think what’s hard about our patient population is that they don’t always have the same phone number, and so it’s really hard to get in touch with them. But then, they really don’t have the resources to make appointments for follow-up of all your tests, and they can’t take off work, and they don’t have transportation to get here. -OB/GYN, Residents’ Clinic*

Providers were also challenged in repeating syphilis screening in the third trimester due to women not keeping prenatal care appointments. To combat this, the clinics had a process to follow when women “no show”:

*If we have patients that don’t show, our front desk person calls them after they miss the appointment. They get a phone call two days before the appointment, they get a phone call one day before the appointment and once they missed the appointment they get a call another phone call to figure out why. We’ll try to call them three times mostly and we’ll send them a letter if we hadn’t heard from them still. Because a lot of people change their numbers, frequently. -OB/GYN Residents’ Clinic*

### Lack of follow-through with partner testing

Many of the providers voiced concern over the lack of follow-through to ensure that the woman’s partner was treated. Providers reported spending time teaching women about the importance of partner testing and treatment, especially as related to a healthy pregnancy. Typically, partners are not treated at the prenatal clinics; therefore, providers have no confirmation that treatment is done.

*I mean we can tell them until we’re blue in the face, that their partner needs to be treated but we have almost no way to follow that up or have evidence that it actually happened. And we know out of anecdotal stories of “oh he went to this place and they told him x,y, and z” and it might not all be total falsities. I have no idea. It’s just frustrating that we can’t close that loop, unfortunately here.* -*OB/GYN, Residents’ Clinic*

The Louisiana public health clinics that provide free treatment were mentioned as the most likely place to refer partners, in spite of awareness that there had been closures and reduced days for availability of care.

*I know the public health system here doesn't give as much access for treatment as it maybe could. And so we always have an uphill battle always trying to get people to treat their partners.* -*OB/GYN, Residents’ Clinic*

Only the providers at the federally qualified clinic indicated they provided a clinic-based avenue for partner treatment.

*I, as an OB/Gyn, don't typically do partner testing, but our system can. So we refer the WHOLE UNIT [Woman and Partner] for treatment to our primary care division, which can treat everybody. -OB/GYN, Federally Qualified Community Clinic*

Providers also voiced frustration over the official state reporting process for STDs. All believed they and their agencies followed state requirements for reporting, but wished they had some confirmation that the process was working.

*It’s more about AFTER the treatment of the patient, and that’s our struggle. Who follows up? How do we get them to make certain that they [partners] have taken their medicine? That the woman’s, their contacts have been taken? It gets convoluted between local health departments, state health department. Who is following this? -OB/GYN, Residents’ Clinic*

*We fax and we get nothing faxed [back]. Well, I guess that’s reported? I hope that works out. And there’s often questions of: Who’s obligated? Is the lab reporting? Do WE report? That always comes into the explanation as well. –OB/GYN, Private Clinic*

### Need for community connection for prevention

Providers agreed more community involvement was required to address the barriers to community-wide prevention. Most providers were fairly certain the preventive health messages would be better received if coming from members of the women’s community.

*Honestly, I think prevention starts within the community. From community leaders, women in the community – not people that are elected or appointed, but literally the one person who everybody goes to in the neighborhood, to ask questions and getting them empowered to help educate other women. - OB/GYN, Residents’ Clinic*

*We can talk about it all day long, but unless you have someone that can relate to the patient on a day-to-day basis and can empower them about their sexual practices, the consequences of STIs—then, it’s going to be difficult to make a HUGE difference. Not impossible; I think I’m all for starting this process and doing anything we can to help by putting all of our support behind. But just in my experience, I feel like … it’s people within the community that are going to need to really step up as the … the guiding force in a lot of this. -OB/GYN Residents’ Clinic*

This provider saw the need for larger societal changes necessary to see a significant difference in community prevention.

*This sounds so old-fashioned but we have to get the word out that sex can be very bad and dangerous … In the home, when children see their parents misbehaving, guess what they're gonna do?... So if the parents have multiple sex partners and doing drugs, not a good environment for these children to grow up in. So they don't know any better. And this is not just African American, this is Caucasian, this is all races. It is a local problem, it is a national problem. And abstinence needs to be discussed … . It's got to be part of the discussion, I think we can do a better job with that. -OB/GYN, Residents’ Clinic*

A couple of providers suggested changes that aimed to individualize care and improve access at the community level. One suggested “case management” for high-risk women and another called for development of “community perinatal clinics.”

*Community perinatal clinics have been effective in the past of helping with these types of issues. And these clinics need to be in impoverished neighborhoods where people do not need transportation to get to them. I think it's preferable if you can have group classes and searching for the poorest neighborhood in East Baton Rouge, and have funds to go to a community center so we have education classes for pregnant women and you can encourage 6, 7, or 8 of these young ladies to go to the class at the same time. And through that process you could say, "Well, are you aware that a sexually transmitted disease or syphilis, which many people know about, could damage your baby's heart? Damage your baby's teeth? Make it grow small? And cause even stillbirth?" That would probably—that would be most bang for your buck there. –Maternal-Fetal Medicine Specialist, Private Clinic*

Similarly, another provider talked about there being a “*disconnect with some of our patients*” and believed more was needed to improve relationships and change the image of providers in the community.

*I think there’s so much stigma about medical care, and not unique to Baton Rouge but definitely present in Baton Rouge, and about doctors only wanting to take my baby away, they’re only going to get me in trouble, they’re only just going to tell me things I don’t want to hear, they’re not going to help me, I don’t need prenatal care, there's no benefit, there’s nothing wrong with my baby, all these things. If there was just more a collaboration, instead of an us against them thing. If they thought of our clinic as actually partners instead … -OB/GYN, Residents’ Clinic*

The provider went on to describe how she and obstetrical residents connected with a community activist to improve this:

*That was literally the theme of it, how can we do this better. The woman who came was incredible, she was awesome and she had so many insights. She was very motivated to help women in her community … She was in her early twenties and has two kids. Both delivered here. … She agreed that if we could be more present and be more out in the community then that would benefit everybody. -OB/GYN, Residents’ Clinic*

The provider stated she had *“tried to get into the school to talk to the kids- things likes STDs and birth control, whatever. And we got no response whatsoever, literally none from a single school in the whole area.”* In spite of this roadblock, she saw the need for prenatal care providers to continue to work on changing relationships with the community.

Providers suggested several community-based educational strategies, including billboards, social media, and television. Face-to-face outreach was also recommended, if done with sensitivity:

*Educational seminars, but whether they attend or not is a problem. It has to be done in the particular area where people live. Trying to get people, for example to come here, with transportation issues is just not gonna work. You have to go to them, instead of them coming to us … .. So aside from social media and television, if there was a gatherin'—I'll just call it a gatherin', I don't want to call it a meeting or a conference or whatever—I want to call it a 'gatherin," where information is gonna be provided, testing can be done, and free stuff given. Patients come to that. Patients like free stuff. -OB/GYN, Federally Qualified Community Clinic*

Although providers emphasized the importance of community-based interventions and partnering with the community, not everyone could identify specific local organizations to work with. Those identified included “Title X”, Louisiana Office of Public Health, March of Dimes, and WIC.

### Focus groups

Forty-two women in East Baton Rouge Parish participated in one of the three focus groups comprised of 15, 12, and 15 women respectively (Table 2). Two women were postpartum, while the remaining 40 were currently pregnant and in prenatal care. All group members identified as Black/African American except for one who indicated she was White/Hispanic. The women were predominately low income with 29 (69%) indicating income less than $15,000/year.

**Table 2:** Description of focus group participants (pregnant and postpartum women)

Ages	
18–29	98% (41)
30–39	2% (1)
Childbearing Status	
Currently Pregnant	96% (40)
Delivered past 18 months	4% (2)
Racial Background	
White or Caucasian	0% (0)
Black or African American	96% (40)
Hispanic or Latino	4% (2)
Income	
< $15,000	62% (26)
$15,000- < $20,000	24% (10)
$20,000- < $25,000	8% (3)
$25,000- < $35,000	2% (1)
$35,000- < $50,000	4% (2)

Themes emerged that represented participants’ common experiences around health care during pregnancy: (1) Getting health information about pregnancy, (2) Barriers to prenatal care, and (3) Lack of knowledge about congenital syphilis, testing and prevention.

### Getting health information about pregnancy

Women expressed concern about having a healthy pregnancy and talked about what resources they used when seeking health information. Online, web-based resources were most frequently used for routine pregnancy-related information. All the women had access to the internet via smart phones as well as home laptop computers. Google searches, YouTube and other apps were identified as sources of education and reassurance:

*I use two apps. I use the Pregnancy Plus app, which is one of my favorites because it has a 3-D model on how the baby is supposed to look like and when it develops hair and nails. It lets you know what weight the baby is supposed to be and I feel like it’s accurate because on the app when it says it’s a certain amount of pounds or ounces and I go to the doctor and they tell me that's the weight my baby is. I also use the Baby Center app because it lets you know the size of your baby compared to fruit. And they answer many questions from having symptoms, to what to expect, to what your days are gonna be like, expectations and the future - like how to set up a car seat.*- *Focus group 3 participant*

Some women voiced more satisfaction in apps that provided a more personal connection where they could talk to other women in similar situations, compared to websites without the interactive component.

*I use the Baby Center, the app, you can blog. You can put things on there. They also have other mothers that communicate with you on that app. There’s real life people where you can ask a question, and another mother might relate to you and answer your question. You two can communicate on certain things. Maybe she’s been through it, maybe it’s your first pregnancy, or even your second. But it’s things that you can connect on.-Focus group 2 participant*

Regarding trusting online information, women identified ways to ensure accuracy of content, as narrated by one participant, “*Always ask your doctor what you find online, and they’ll let you know if it’s true or not.*” Another participant suggested looking at the bottom of the web page, to see if it was written by a medical person, and warned that information in blogs can be wrong.

Other women mentioned diverse resources for information, including Planned Parenthood, WIC, social services, and their health care providers.

*WIC helps a lot … Even before you get your vouchers, nowadays you have to sit in a class. They speak to you about health and what you should eat, and what things are in food that you’re gonna get in your voucher, speak with you on certain things. They really bring mothers up to date.-- Focus group 2 participant*

Although health care providers were identified as good sources of information in some cases, accessing that information could be difficult.

*I feel like it’s much HARDER because you have to go through someone. For example, trying to call my health care provider, said you have to go through all the automatic settings, and everything like that. It gets really irritating cause you don’t get the information that you REALLY want. You listen to the audio machine, and they don’t have the answers that you really need.* -- Focus group 2 participant

*You have to wait so much time. If you call your doctor, your doctor might be delivering someone else’s baby, so you might have to wait 1 hour or 2, even if it’s something serious that you need help with. That’s why you use Google. --Focus group 2 participant*

Others felt their doctor or midwife was still the best source of information. There was consensus that it is always better to follow up and have a face to face discussion, even after getting printed information.

*It’s more comfortable when they actually speak to you. You can ask questions directly to the doctor. -- Focus group 1 participant*

### Barriers to prenatal care

#### Delayed access to starting prenatal care

The women overwhelmingly believed that negotiating the state’s Medicaid insurance system was a deterrent to starting prenatal care. As one explained,

*If you call the doctor to make an appointment, they will ask you, “What type of Medicaid do you have?” They are going to, ask for your ID number to CLARIFY first that your Medicaid is active. And once it’s active, then they will make your appointment. If they don’t accept it, they won’t even schedule your appointment. Focus group 2 participant*

Sometimes women had problems matching the prenatal care provider with the correct type of Medicaid insurance, with women stating they were told by providers’ offices that *“we don’t accept that Medicaid health plan,”* or *“Oh, the doctor’s not accepting any patients right now.”* It was the initial approval process, however, that was most problematic. As one stated, *“Because you’re pregnant, it’s supposed to be maybe, like, 48 hours to— approve. You end up waiting as long as like 30-45 days*.” Another explained the need for individual persistence.

*It sometimes takes forever for you to even sign up online. This is my first pregnancy so I signed up for Medicaid right AWAY. And … I got a call from the hospital, they told me that my Medicaid wasn’t active even though I did my Medicaid application. I was able to call Medicaid and stay on them about. I literally had to CALL them and BUG them over and over like “Hey, look. My doctor’s appointment is such and such day.”- Focus group 2 participant*

Another woman already in her second trimester, described her difficulty,

*I was about 16-17 weeks when I found out being pregnant. They actually took them a MONTH before they even gave me my Medicaid. I had to go to an emergency room and a free health center to do pregnancy testing and everything. -Focus group 2 participant*

Women were also aware of other timing barriers in the community, such as doctors’ preferences:

*Some doctors don’t take you after the first trimester. Either you have to go in early, there are only a few that will take you after. With my first one, I was too far along.* -*Focus group 1 participant*

*My last pregnancy ___ actually turned me away at 33 weeks. It was because of my insurance. That’s how I ended up at ____, and you know, after a while my insurance kicked in, but I didn’t see a doctor until about 30-something weeks with my last pregnancy. - Focus group 2 participant*

### Lack of transportation

Lack of transportation was the second most common barrier to prenatal care. A few clinics or Medicaid services provided transportation, but this was sporadic. There was a consensus that improved transportation was needed.

*Transportation does help especially if you don't have your own transportation. That helps you get to doctor's appointments, WIC appointments, whatever type of appointment you have that requires transportation, it comes in handy.* -*Focus group 3 participant*

### Lack of knowledge about congenital syphilis, testing and prevention

All of the women recognized syphilis as a sexually transmitted disease similar to gonorrhea and HIV. Beyond that, there was little consensus on how syphilis affected a pregnancy or the baby.

*Um, since the baby is in a sack, I feel like the baby is protected from a STD that a female can carry*. *-- Focus group 3 participant*

*I have had EDUCATION on syphilis and stuff in the past. But not … what it can do to the baby. I just know if you’re not pregnant, it can make you sterile or you won’t be pregnant because of the medications they give you. As far as pregnancy, no doctors have discussed … pretty much ANY STD with me*. *-Focus group 2 participant*

*It gets transmitted through, BIRTH. That’s the only time it can actually be transmitted, like, through birth. That’s why they schedule C-sections. -Focus group 2 participant*

The women knew they underwent routine prenatal tests during the first visit, and at other times, but most were unsure what the specific tests were and if syphilis screening was part of that. One exception reported good rapport with her midwife:

*When I first went to my midwife she gave me a packet … and it told me all the things they’ll be testing for when they draw your blood in your first trimester, your second trimester, third trimester, and what it was for. -Focus group 2 participant*

Other women who underwent prenatal testing assumed their providers did not discuss syphilis with them because the test was negative; therefore, they didn’t ask about the test or if it would be repeated.

*I think we should be given more information. All we know is, when you go to the doctor, you get tested. You got to be testing negative, cause the doctor is not telling you ANYTHING about it, or anyway to PREVENT it.-- Focus group 2 participant*

*I think MOST of us in here probably didn’t ask questions, cause they didn’t come back and say, Oh, you’re positive. --Focus group 2 participant*

All the women were aware that prevention of syphilis, like other STDs, required *“safe sex”,* further explained by participants as “*using condoms,” “not sleeping with different partners,” “having no sex,”* and *“getting tested with your partner.”* They were aware of the societal issues that increased risk in the local community:

*A woman can be honest and don’t cheat, and the man can be out there cheating behind your back. That’s how some women get it. Men go out there and cheat you. - Focus group 1 participant*

*We also have a lot that is just … what’s the word, down low. If you believe a man is doing something, you’re gonna believe—he’s probably with another woman, you won’t believe it’s more so with a man where … . I think the diseases come especially from where we’re at now, cause we have more men with men nowadays. - Focus group 1 participant*

*Because we have a lot of—junkies. Drug abusers, and, a lot of them know each other. They sit in a circle, in the same house with each other, probably sharing the same needles, or whatever like that—in MY neighborhood, PERSONALLY, from EXPERIENCES, I feel like it’s … THERE. - Focus group 2 participant*

Women thought more should be done to raise community awareness about prevention, testing, and treatment. One participant recommended the messages should be, “Save your life. Save your child’s life, and your partner.”

## Discussion

In this project, we aimed to identify gaps in knowledge and barriers to accessing treatment and screening for congenital syphilis. Our investigation with health care providers and community members found several common themes. Doctors and community women agreed that syphilis was a problem in the area. Several barriers to prenatal care were identified, transportation being notable, and women particularly identifying difficulties in accessing Medicaid. Lack of ability to follow up the partner was a consistent issue. Knowledge of congenital syphilis was not high in the community, although some of the basic facts – that it was sexually transmitted and that it was tested for in pregnancy – were widely known. Both women and providers were describing their general experience, rather than two sides of the same interaction, so they cannot be directly validated against each other. However, in some cases, perceptions of barriers to treatment differed, based on the day-to-day experiences of the two groups: providers were more likely to identify issues around contact with patients, while community women were more likely to identify issues around signing up for Medicaid and enrolling in prenatal care.

Many of these factors have been highlighted in previous studies, both in the U.S. and abroad. A previous study in Caddo Parish in northern Louisiana conducted interviews with clinicians, community organization representatives, and women of childbearing age [15]. This study also mentioned the patchwork provision of health care; difficulty in finding prenatal care, especially later in pregnancy; and transportation and work and child care issues that made getting to appointments difficult. More strongly emphasized than in our study were lack of sexual education and awareness, low perceived need for prenatal care in non-emergency situations, and difficulties around accessing the drug. Benzathine penicillin was not always stocked, not completely reimbursed, and the protocol requires multiple injections [15]. A qualitative review of congenital syphilis cases in Indiana found that a large majority of mothers were insured and that the providers were following screening recommendations; however, many women had insufficient number of prenatal care visits and often had social vulnerabilities (such as homelessness) [16].

Other studies in substantially different contexts have identified similar barriers. A qualitative study in the Democratic Republic of the Congo and Zambia [17] found these included, at the pregnant women’s level, late enrollment in prenatal care, lack of knowledge about consequences and treatment of syphilis, and stigma. At the providers’ level, these included lack of knowledge and training about best practices and reservations regarding same-day screening and treatment. At the system level, these included fragmentation of the health system, poor accessibility of clinics, and staff and product shortages [17]. Studies of clinicians in Brazil identified as barriers the lack of attendance of the partner, late prenatal care, delayed results and lack of return by the woman, and difficulties in administering benzathine penicillin. Knowledge by the clinicians was limited on some factors as well [18–21]. A study in Ghana identified availability of test kits and personnel training as key clinician-side predictors of screening, while attending a public hospital, being willing to request screening, and being later in pregnancy were associated with uptake of screening among pregnant women [20].

Strengths of the study include the systematic interviewing, the provision of transportation when needed, and the inclusion of both community members and health care providers. Limitations include the focus on a single geographic area and the lack of specific inclusion of affected patients. In addition, the study recruited through organizations that provided benefits to pregnant women and families – women who were completely detached from the system, or incarcerated, were not included, and may be some of the most vulnerable. Focus groups were conducted only in English – in some parts of the country, non-English speakers would be a group of major concern.

This study suggests possible recommendations, at different levels (Table 3). Of course, all interventions should be evidence-based and ideally rigorously tested relative to controls, as well as reflecting the realities of clinician’s and high-risk pregnant women’s lives [22, 23]. Interventions most readily amendable to change at the individual and community levels should be assessed and prioritized. For example, at the individual and community level, strategies to promote better medical and sexual education could improve knowledge of the risks and treatment availability for syphilis. Such programs have proven effective for decreasing other STDs, as well as risky sexual behavior [24, 25]. In the current clinic system, immediate changes to provide more extensive follow-up procedures for the woman and her partner can be evaluated and planned. In some situations, expedited partner therapy has been shown to be effective [26]. Such procedures should be designed with care; partner notification may be a problem if the woman fears domestic violence, for instance [27]. For more widespread clinic changes, the medical professional can be targeted for education for acceptability of using the rapid, accurate, point-of-care tests to limit the loss to follow-up caused by lag in services and labs in different locations. Such tests improve outcomes for HIV-infected women [28], and our implementation trial in Africa found that a multifaceted behavioral intervention improved the likelihood of immediate treatment after diagnosing syphilis in pregnancy [29]. At the societal level, structural barriers need to be addressed; in a narrow focus, this includes easy access to Medicaid [30], widespread acceptance of all forms of Medicaid, and access to transportation for prenatal visits [31]; in the wide focus, housing, substance abuse prevention and treatment, and economic stability allow for prioritizing prenatal care.

**Table 3:** Social-Ecological Model Levels and Potential Strategies to Address Congenital Syphilis

SEM Level	Potential Strategy	Supporting comments
**Individual-level**	• Increase knowledge about risk from congenital syphilis • Increase perceived effectiveness of screening and treatment of syphilis especially early in pregnancy	• *“About 30% of them initiate their care* ***…*** *well into their second trimester. And I’d say another 30% in their third trimester or no care at all.”(provider)* • *“My last pregnancy ___ actually turned me away at 33 weeks. It was because of my insurance.”(community woman)*
**Social and sexual network**	• Address barriers to partner testing and assist persuading male partners to prevent reinfection • Track all sexual partners for syphilis case patients	• *“We can tell them … that their partner needs to be treated but we have almost no way to follow that up or have evidence that it actually happened.” (provider)* • *“A woman can be honest and don’t cheat, and the man can be out there cheating behind your back.” (community member)*
**Community**	• Provide education and support to prenatal care providers • Accept walk-ins for initial pregnancy diagnosis and screening tests • Simplify STD testing • Comprehensive case manager • Confidential services to help women seeking services • Transportation service • Utilize text messages and apps to better inform pregnant women and assist their access to services • Provide flyers after in-person consultation • Host community events to educate and screen both men and women in the community	• *“We fax and we get nothing faxed [back]. … And there’s often questions of: Who’s obligated? Is the lab reporting?” (provider)* • *“If you call the doctor to make an appointment, they will ask you, “What type of Medicaid do you have?” … If they don’t accept it, they won’t even schedule your appointment.”(community woman)* • “*We usually have to call them to come back in to get treated. And so some of them actually don’t return” (provider)* • *“Community perinatal clinics have been effective in the past of helping with these types of issues.” (provider)* • *“Trying to get people, for example to come here, with transportation issues is just not gonna work.” (provider)* • *I use two apps. I use the Pregnancy Plus app … I also use the Baby Center app … they answer many questions. (community woman)* • *“I think we should be given more information.” (community woman)* • *“So aside from social media and television, if there was a gatherin’— … where information is gonna be provided, testing can be done, and free stuff given.”(provider)*
**Policy/Enabling Environment**	• Post billboards and ads to raise awareness in the community • Correct administrative barriers in Medicaid that delay accessing prenatal care • Offer in-class information sessions	• Providers suggested several community-based educational strategies, including billboards, social media, and television • *“It sometimes takes forever for you to even sign up online.”(community woman)”* • *“Educational seminars, but.. it has to be done in the particular area where people live.” (provider)*

## Conclusions

Although congenital syphilis affects a relatively few infants per year, its resurgence points to gaps throughout the system. In this study, both health care providers and community members agreed on the need for easier and earlier access to health insurance during pregnancy, and the involvement of the community in education and outreach.

## Supplementary Information

**Additional file 1.** Screening form and guide: Prenatal Care Providers.

**Additional file 2.** Screening form and guide: Pregnant Women.

## Data Availability

The datasets generated and analyzed during the current study are not publicly available to protect participant confidentiality but are available from the corresponding author on reasonable request.

## Abbreviations

CDC: U.S. Centers for Disease Control and Prevention
STD: Sexually transmitted disease
