# Supplementary material for: Congenital syphilis in East Baton Rouge parish, Louisiana: providers’ and women’s perspectives
Source: BMC Infect Dis. 2021 Jan 13;21:64. doi: 10.1186/s12879-020-05753-6 (PMC7805072; doi:10.1186/s12879-020-05753-6)
Supplement: Supplementary file 2 — Additional file 2. Screening form and guide: Pregnant Women. [file 12879_2020_5753_MOESM2_ESM.docx]

**Screening Form: Pregnant Women**

*To be administered to each potential participant individually, in private.*

Thank you for expressing interest in our study! We are conducting this study to learn more about what women do before pregnancy and during pregnancy to keep their babies healthy. The study will involve one group interview, and will last approximately 1.5 – 2 hours. A focus group is a small group of people who gather to talk together about a topic. You will be compensated with a $25 gift card for your time. I just need to ask you a few quick questions to make sure you are eligible to participate. Please remember that all of your answers will be confidential and we will not share any of this information outside of the study.

Are you interested in continuing?

Yes 🡪 *Continue*

No 🡪 *Exit script*

1. Do you currently live in [Kern County/ East Baton Rouge Parish]?
   1. Yes 🡪 *Continue*
   2. No 🡪 *Exit script*
2. Have you lived in [Kern County/ East Baton Rouge Parish] for at least 6 months?
   1. Yes 🡪 *Continue*
   2. No 🡪 *Exit script*
3. What is your age?
   1. ______

*If under 18 🡪 Exit script*

*If 18 or over 🡪 Continue*

1. Are you currently pregnant?
   1. Yes 🡪 *Continue*
   2. No 🡪 *Exit script*

1. When you think about all the money you make, from all different sources including public benefits, what is your yearly household income, before taxes? When we say ‘household’ we mean anyone who lives in your house and contributes to expenses. *[Prompted]*
   1. Less than $15,000
   2. Less than $20,000
   3. Less than $25,000
   4. Less than $35,000
   5. Less than $50,000
   6. Less than $75,000
   7. $75,000 or more
2. Are you Hispanic, Latino/a, or of Spanish origin?
   1. Yes
   2. No
   3. I don’t know
3. How do you describe your race? You can select all that apply. (*Read answer choices aloud*)
   1. White
   2. Black/ African American
   3. Asian
   4. American Indian / Alaska Native
   5. Pacific Islander
   6. Other (Specify):__________________

***If eligible:***

Great, thanks for answering my questions! It looks like you are eligible to participate in our study! For next steps, we just need to schedule you to participate in a focus group discussion. The focus group will last about 1.5-2 hours, and you will be compensated with a $25 gift card for participating. The focus group will take place in a location convenient to you, like in library or meeting space. Would you like to participate?

Yes 🡪 *Continue to schedule participant*

No 🡪 *Ok, thank you for your time.*

***If NOT eligible:***

Thank you so much for your time. Based on your responses, it appears that you are not eligible for the study we are conducting. Thank you for your time.

**Semi-structured Focus Group Guide for Pregnant Women**

**Introductions:** Hello, my name is _______________________. I have been asked by the University of California / Tulane University to guide this discussion today. First, I want to thank you for taking the time to speak with me today.

We will be discussing your thoughts and ideas about health in general and the health and well-being of pregnant women in Kern County/ East Baton Rouge Parish. We are trying to learn how we can improve antenatal care services so as to meet the needs and interests of pregnant women in your community. Our discussion will provide us with information that can be used to improve our antenatal care in Kern County / East Baton Rouge Parish.

Before we begin, I’d like to explain what a focus group is and then give you some information about this specific focus group. A focus group is like a discussion group. It’s a way of listening to people and learning from them. In a focus group, people are asked to talk with others about their thoughts and ideas about a subject. We want to know what is important to women like yourself in the community. We are interested in hearing what you think and feel about each topic. There are no right or wrong answers. We expect that many you will have different points of view

Our discussion today will take one and a half to two hours. We’ll take a ten-minute break about halfway through. It is best if our discussion is informal. There is no need to wait for me to call on you to respond. In fact, I encourage you to respond directly to the comments other people make. If you don’t understand a question, please let me know. I am here to ask questions, listen, and make sure everyone has a chance to share.

We are interested in hearing from everyone in the group. If someone seems to be stuck on a topic, I may interrupt you. If I do, please don’t feel bad about it, it’s just my way of making sure we get through all of the questions and everyone has a chance to talk.

We will be tape recording the discussion tonight, because we don’t want to miss any of your comments. No one outside of the research team will have access to these tapes. No names will be included in any reports. Your comments are confidential.

Please feel free to speak openly and honestly but we request that after you leave this room you do not share or repeat any information you hear during this focus group. Please make sure that no personal comments leave the room. All of our questions will be about pregnant women like you in your community. We won’t ask anything about you specifically and you will not be asked to speak about your own personal experiences, but you are free to do so if you would like. Everything we talk about will be kept confidential. If there are any questions you do not feel comfortable answering, that is okay and you can skip that question. Does anyone have any questions for me before we begin? May we turn on the tape recorder?

1. **Ice Breaker**
   1. First, so we can all get to know each other a little, let’s go around and have everyone tell us what your due date is and the ages of any other children you may have.
   2. What are the things about motherhood that excite you?
2. **Health Information Seeking**

Now I would like for us to talk about the ways women in your community get information about their health, overall and during pregnancy.

- 1. When pregnant women have questions about their health – in general or related to their pregnancy, where can they go for information? *Probe – The doctor, a friend, a family member, the internet?*
     1. [If not mentioned] How do women feel about seeking information from a health care provider or the health department?
  2. How common is it for women in your community to seek health information online?
     1. Which websites are do they use? *Probe: Website names, Blogs / names of blogs?*
     2. How do you tell if health information you find online is trustworthy?
  3. How do pregnant women prefer to get health information? *Probe – Newspapers, websites, women’s groups, radio shows, discussion with people, video, television, other written information (Pamphlets/brochures, photonovellas [explain story with pictures, fact sheets, patient stories [testimonials]).*
  4. Do pregnant women seek health information related to pregnancy from WIC? Temporary Assistance for Needy Families (TANF)?
     1. If yes, what type of information do they seek here? How do women feel about the information available from WIC / TANF? *Probe: is it reliable? Can they find what they are looking for?*
  5. Have you ever downloaded an app that has pregnancy-related health information?
     1. If yes, which app did you download? How do you like it?

1. **Use of Health Services**

Now we’re going to talk about some things related to general health and pregnancy. Throughout the focus group, whenever we talk about healthcare, please keep in mind that we are referring to care *since becoming pregnant*. Also remember that any answers you give us will be kept completely confidential.

- 1. What are the biggest problems pregnant women face when trying to access healthcare? *Probe – Lack of insurance? Immigration status? Lack of ability to pay? Office is too far away / transportation problems? Cannot get an appointment? Do not have a doctor?*
  2. Do most women have health care coverage, such as health insurance, a prepaid plan such as an HMO, a government plan such as Medicare or Medicaid, or Indian Health Service or other types of State Health Programs?
     1. If no, what are the reasons they do not have health care coverage? Or why do they think they cannot get health care coverage?
     2. Is it harder for some women than others to get health care coverage? IF YES, which women struggle to get health care coverage and WHY? *Probe – Immigrants? Women who speak English as a second language?*
  3. I know women seek prenatal care at different locations and from different providers so each of your experiences is unique. That said, I would like to know your general feelings about the process of getting care while pregnant.
     1. How many prenatal care visits do women usually have during a full term pregnancy?
     2. How easy is it for pregnant women to schedule appointments with their prenatal care provider?
     3. How long do women usually have to wait each time they go to a clinic for prenatal care?
  4. When women have a problem with or a question related to their health, do they feel comfortable talking to any of their health care providers (about the problem or questions)?
     1. If yes, who? If no, why?? (nurses, physicians, midwifes)
     2. With which health professional do pregnant women spend more time?
     3. Are there any health professionals pregnant women try to avoid?

**IF NEEDED/APPROPRIATE – CONSIDER TAKING A 10 MINUTE BREAK HERE**

1. **Prevention Strategies**

During prenatal care it is routine for some health promotion activities to be implemented at health care facilities. Health care promotion activities include tests that are given to promote healthy life styles, and activities that promote health and prevent diseases. For example: tests are often conducted during antenatal care to prevent complications to your health and your baby’s health, such as syphilis, anemia, HIV, proteinuria, etc.

- 1. Have you been given any tests during your pregnancy to prevent some disease/health problem or to promote a healthier pregnancy?
     1. If yes, which tests?
     2. Within the health care team, who was the person responsible for asking you about or performing the tests?
     3. Which is the test you most remember, and why do you think it made a higher impact on you?
  2. Were you given any written information (e.g., brochures, fact sheets) about these tests? How was this information presented? Was it clear?

1. **Knowledge**

Now we’re going to switch to talk about a specific health topic that some of you may have heard of. Just as a reminder, we are not going to ask you specifically about your experiences with this health topic. We are interested in what you and women in your community think. There are no right or wrong answers, we are just interested in getting your honest thoughts and opinions.

- 1. What do you know or what have you heard about syphilis? What about syphilis during pregnancy?

**SYPHILIS DEFINED:** So we are all on the same page, syphilis is a sexually transmitted infection that can cause serious health problems if it is not treated. Syphilis is divided into stages (primary, secondary, latent, and tertiary). There are different signs and symptoms associated with each stage.

- 1. Please tell me what you know about the consequences of syphilis during pregnancy on the health of your baby and on your health? *Probe (if not mentioned): Can syphilis be passed from a woman to her unborn baby?*
  2. If a pregnant women is infected with syphilis, what do you think the benefits are to her of being diagnosed during pregnancy? What are the benefits for her baby? At what time during the pregnancy (first, second, third trimester) do you think she should be tested?
  3. From your recollection, have you been tested for syphilis during a routine prenatal care visit during this pregnancy?
     1. How were you tested?
     2. How did you feel about it?
     3. Were your questions answered (if you had any)?
  4. Other than being tested for syphilis by a health care provider, do you think there are other ways you can tell if you have the infection, such as by the presence of symptoms?
     1. What are some symptoms of syphilis?
     2. Do you think it’s possible that a woman might experience no symptoms even if she has syphilis?
  5. Do you think a pregnant woman with syphilis can receive treatment to cure the infection (while she is pregnant)?
  6. How can people protect themselves from getting syphilis or transmitting it to others?
  7. Do you feel that women in your area are at higher risk of getting or having syphilis, compared to women throughout the rest of the state? The rest of the United States?
  8. What do you think are the most important messages for women like yourself to hear about the benefits of being tested for syphilis and receiving treatment if she is positive?

**Conclusion:** I would like to thank you all for your participation in this focus group! I appreciate the time you have taken to answer all of my questions and share your feelings with me and the group. I have learned a lot from you. Before we end, do you have anything else you would like to share or any questions you would like to ask me? Thank you!
